# Dysregulated neutrophil function in individuals with alpha-1 antitrypsin deficiency caused by modified membrane cholesterol content.

**DOI:** 10.1186/1753-6561-9-S7-A7

**Published:** 2015-10-27

**Authors:** Bakr Jundi, Michelle White, Noreen Lacey, Noel G McElvaney, Emer Reeves

**Affiliations:** 1 Dept. of Medicine, Respiratory Research, Beaumount Hospital, Dublin 9, Ireland

## Background

Individuals with alpha-1 antitrypsin (AAT) deficiency (AATD) are predisposed to early-onset emphysema and neutrophils are the primary effector cells responsible for the pathological manifestations of AATD lung disease. As AAT interacts directly with the circulating neutrophil membrane [1], the question that this project addressed was: are AATD neutrophils structurally and functionally altered? The aim of this study was to explore a link between disrupted membrane structure and impaired trafficking of cholesterol in AATD neutrophils.

## Methods

Circulating neutrophils were purified from blood of patients with AATD and from healthy control individuals (n=7). Membranes and cytosols were isolated from neutrophils by sucrose-gradient ultracentrifugation. Cholesterol and calcium levels were fluorometric quantified and calpain levels measured using a calpain activity assay. Caveolin-1 expression was examined by Western blot analysis. Statistical comparisons were performed by Student's t-test.

## Results

Neutrophil cytosols of AATD individuals had increased calcium concentrations (n=7, p=0.04) and activation of the calcium dependent protease calpain (n=7, p=0.01). Furthermore, levels of the cholesterol trafficking protein caveolin-1 were significantly lower in AATD neutrophil cytosols (n=6, p=0.01) leading to significantly decreased membrane cholesterol content when compared to healthy control cells (n=5, P=0.045).

## Conclusions

In summary, our findings have demonstrated for the first time increased calcium, increased calpain activity causing proteolytic cleavage of caveolin-1, and decreased membrane cholesterol content of AATD neutrophils. This novel data may in part explain the dysregulated activity of this innate immune cell in AATD.
